# Level of Dyspnoea in Patients with COVID-19 in Poland

**DOI:** 10.3390/ijerph191912203

**Published:** 2022-09-26

**Authors:** Monika Gałczyk, Anna Zalewska, Sławomir Chlabicz, Bożena Ewa Kopcych

**Affiliations:** 1 Department of Physiotherapy, Faculty of Health Sciences, Łomża State University of Applied Sciences, Akademicka 14, 18-400 Łomża, Poland; 2 Department of Family Medicine, Medical University of Białystok, Mieszka I 4B, 15-054 Białystok, Poland; 3 Department of Nursing, Faculty of Health Sciences, Łomża State University of Applied Sciences, Akademicka 14, 18-400 Łomża, Poland

**Keywords:** Medical Research Council, COVID-19, adults, Poland, dyspnoea

## Abstract

Objectives: The study aimed to assess the level of dyspnoea during the COVID-19 pandemic in Poland. Methods: The online questionnaire was conducted among 204 Polish adult respondents with a positive SARS-CoV-2 test result. The level of dyspnoea was assessed by the modified Medical Research Council (MRC) Dyspnoea Scale in Polish. Results: Dyspnoea is most common in patients with severe COVID-19, and the prevalence of dyspnoea in the study population of patients with COVID-19 was low (34% respondents presented with dyspnoea with a score of 1 or higher). Conclusions: There is a need for further investigation and close monitoring of the extent of dyspnoea in different social groups, especially in the event of a prolonged pandemic and the emergence of further waves of COVID-19.

## 1. Introduction

COVID-19 disease, defined as acute respiratory distress syndrome, was first detected in 2019 in Hubei Province, Wuhan. The WHO declared COVID-19 as a public emergency of international concern [1,2,3]. All the changes that were implemented worldwide (such as isolation, the change in the form of work and study from stationary to online, restrictions on movement, etc.) were aimed not only at reducing the spread of the SARS-CoV-2 virus but also its consequences: primarily the exacerbation of the course of various chronic diseases as well as deaths [4,5,6,7].

The severity of the condition has been divided into four levels. The first group are asymptomatic patients, the second group are symptomatic patients who are isolated at home, the next group are symptomatic cases admitted to hospital and the most severe group are those requiring medical intervention—mainly ventilation [8]. The most common clinical symptoms of patients with COVID-19 infection include fever, sore throat and muscle pain and cough. In more severe cases, the elderly and people with other comorbidities may present with the symptoms of respiratory failure, and heart or kidney damage [9].

There are many definitions of dyspnoea in medicine, but it is most often presented as a subjective sensation of shortness of breath or difficulty in breathing. It is a frequent and distressing symptom reported by patients. It depends not only on physiological or environmental factors, but also on psychological factors such as anxiety during a dyspnoea attack or social factors such as the threat of losing work or social activity. In healthy people, dyspnoea may occur during heavy physical exertion but is treated as a normal reaction, whereas in patients it may occur at low exertion or even at rest [10,11,12].

Dyspnoea usually develops 7 or 8 days after the onset of COVID-19 symptoms and is typical in patients with severe SARS-CoV-2 [13,14,15]. Several studies in the literature show that both dyspnoea and cough are the most common symptoms among patients with a mild course of SARS-CoV-2 infection [16,17,18,19,20].

This study aimed to assess the level of dyspnoea during the COVID-19 pandemic in Poland. The main objective was to find if there is a correlation between the severity of infection, the need for hospitalisation and level of dyspnoea, and if there is a correlation between sex, age and the occurrence of dyspnoea in patients with COVID-19.

## 2. Materials and Methods

### 2.1. Participants and Procedure

In August 2021, at the time of the COVID-19 pandemic, Polish adult residents with a positive SARS-CoV-2 test result participated in an online cross-sectional questionnaire. The researchers distributed the survey as well as the information about it on the Facebook profile of the Lomza State University of Applied Sciences providing a link to a Google form. Additionally, mails with a link to the questionnaire were sent to employees and students of the University. For the study the ten most frequent symptoms were chosen, that is: fever, cough, myalgia, olfactory and taste disturbances, fatigue, general weakness, diarrhoea, dyspnoea or shortness of breath and headache [21]. As there is currently no categorization of SARS-CoV-2 symptoms available in the literature, the authors came up with their classification of symptoms (severe, fully symptomatic, oligosymptomatic, asymptomatic). Respondents filled in the questionnaire following the instructions provided by the authors. Infection deemed as fully symptomatic COVID-19 meant having at least six of the ten most frequent symptoms (fever, cough, myalgia, olfactory disturbances, dysgeusia, general weakness, fatigue, diarrhoea, difficulty breathing or dyspnoea, headache) present. The presence of up to five of the ten most common symptoms (apart from breathing problems or dyspnoea) was labelled oligosymptomatic and with no symptoms was classified as asymptomatic. Additionally, the respondents stated whether they were hospitalized. Respondents who were hospitalized for COVID-19 were considered as severe cases. The questionnaires that did not meet the study’s inclusion requirements were removed from the pool. Inclusion conditions included: an age of at least 18 years, consent to participate in the study and a SARS-CoV-2 positive test result. Those who were under 18 years of age, did not give informed consent and did not have a positive SARS-CoV-2 test result were excluded from the study. 

The project was approved by the Senate Committee on Ethics in Scientific Research of the University of Medical Sciences in Białystok, KB/162/2020/2021.

Participation in the study was voluntary and the results were published in accordance with Regulation (EU) 2016/679 of the European Parliament and of the Council of 27 April 2016 on the protection of natural persons with regard to the processing of personal data, on the free movement of such data, and repealing Directive 95/46/EC, in the Personal Data Protection Act of 10 May 2018 (Journal of Laws 2018, item 1000). (General Data Protection Regulation). The respondents were instructed on the objectives of the study, the way the poll was conducted and the applicable data protection laws.

### 2.2. Methods of Assessing Dyspnoea

One of the most used scales in medicine to assess the severity of dyspnoea is the Medical Research Council (MRC) Dyspnoea Scale. There are 5 questions on it: 0 (dyspnoea only with exertion), 1 (shortness of breath occurs when walking fast on flat terrain or climbing a small hill), 2 (due to shortness of breath, the patient walks slower than peers or has to stop to catch his breath when walking at his own pace on flat terrain), 3 (after about 100 metres or after walking for a few minutes on flat terrain, the patient has to stop to catch breath), 4 (dyspnoea at rest, making it unable to carry out daily tasks independently or leave the house) [22]. For Polish circumstances, the original scale was modified [23].

### 2.3. Statistical Methods

The Kruskal–Wallis test was applied to check whether the differences between the groups were statistically significant; the researchers chose this statistic test because of the asymmetry of the variables under study. The Spearman’s rank correlation coefficient was used to investigate the relationship between the age and dyspnoea of the participants. The Mann–Whitney test was used to investigate the relationship between need for hospitalisation and level of dyspnoea. In all statistical analyses, a *p*-value significance threshold of 0.05 was used.

## 3. Results

### 3.1. General Characteristics

Data from 204 (122 females and 82 males) people were used for the statistical analyses (Table 1). The respondents were predominantly females (59.8%).

More than 90% of respondents had not been hospitalised for COVID-19 (Table 2). The largest proportion of respondents with COVID-19 was oligosymptomatic (48.5%). Only 25% of the people rated the symptoms present during the illness as symptomatic and severe (Table 3).

### 3.2. Level of Dyspnoea—MRC Scale

**Figure 1:**
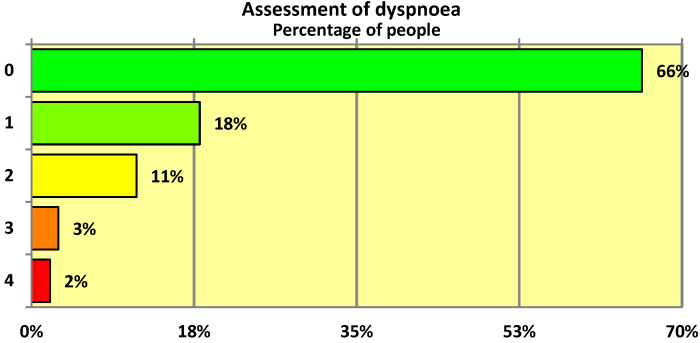
Dyspnoea level of subjects who underwent SARS-CoV-2 virus infection.

The 5-point MRC scale was used to assess the degree of dyspnoea in individuals who had undergone SARS-CoV-2 infection. Overall only 34% of respondents presented with dyspnoea with a score of 1 or higher on the modified MRC scale. As can be seen, the mean degree of dyspnoea was rather low at 0.57 (on a scale of 0–4 points) (Table 4). The median was 0 points (Table 4), which means that more than half of the people who had undergone COVID-19 infection had no dyspnoea at all—as can be seen from the graphical representation of the MRC scale distribution (Figure 1), almost exactly two-thirds (66%) of the respondents had no complaints of this kind.

### 3.3. Course of Infection and Level of Dyspnoea

Levels of dyspnoea measured in the study are summarised in Table 5. There are statistically significant differences in dyspnoea level, with the best results in those who had an asymptomatic course of SARS-CoV-2 infection, worse in those with mild symptoms and the worst in those who were fully symptomatic or had severe infection.

### 3.4. Need for Hospitalisation and Level of Dyspnoea

In addition, those who were hospitalised had higher levels of dyspnoea than those who were not hospitalised for COVID-19 (Table 6).

### 3.5. Age and Sex and Dyspnoea

There were large differences between men and women in the level of dyspnoea (Table 7). This was partly due to the large differences in the mean age between men and women with women being significantly older.

The relationship between age and dyspnoea was examined separately for the female and male groups. We found statistically significant correlations between age and dyspnoea in both groups, but the correlation was stronger among males than among females as expressed by Spearman’s rank correlation coefficient of 0.57 (*p* = 0.0000) among males and 0.21(*p* = 0.0198) among females.

## 4. Discussion

In patients with COVID-19, dyspnoea is one of the predominant symptoms at the onset of the disease, and dyspnoea increases with the severity of pneumonia [24]. The authors decided to use the Medical Research Council (MRC) Dyspnoea Severity Scale for their study, following the recommendations of the Polish National Chamber of Physiotherapists on the instruments to be used when examining patients with respiratory disorders and according to COVID-19 management guidelines [25].

The authors’ preliminary data suggest that the prevalence of dyspnoea in patients with mild COVID-19 is low. These results are consistent with the study by Ora et al. [26]. Chinese hospitalised patients with COVID-19 were 27.3 percent free of dyspnoea, and 31.3 percent had dyspnoea when walking fast on flat terrain or climbing a slight hill [27].

In our study, the highest dyspnoea scores were obtained by patients who were hospitalised. Italian post-acute COVID-19 patients suffered from dyspnoea even during the simplest activities [28]. In a study by Guan et al., the prevalence of dyspnoea was higher in patients with more severe COVID-19 [2]. Data from Tleyjeh et al. [29] are similar. A UK study of 327 hospitalised patients with SARS-CoV-2 infection enrolled in a prospective multicentre cohort study at least three months after hospital discharge found that 47% reported an increase of at least one level on the MRC dyspnoea scale [30]. These symptoms were largely independent of age, which differs from our study, which found a statistically significant correlation of dyspnoea with age. Furthermore, the high frequency and severity of symptoms in patients with severe COVID-19 highlights the long-term impact on population health and well-being [30].

The advantages of the survey were: easy access to the study groups and minimal cost. The study also had certain disadvantages, such as the subjectivity of the responses and the small size of the group being polled. Consequently, it was hard to examine the prevalence of dyspnoea at the national level. Moreover, the respondents were not asked about the standard procedure for identifying the SARS-CoV-2 virus. Instead, they were just asked if they had a test conducted and what the result was. Additionally, since the survey was performed online, only those with an internet connection had access to the study link. Confounding variables also had an impact on the study. Because the study was observational, a causal link between dyspnoea and COVID-19 cannot be established.

## 5. Conclusions

The study confirms that dyspnoea is most common in patients with more severe COVID-19 and that the prevalence of dyspnoea in the study population was low (34% respondents presented with dyspnoea with score of 1 or higher). There were also statistically significant correlations between the level of dyspnoea and age: the older the age, the higher the score of breathlessness. The small sample size does not allow for conclusive results and further research is needed. Further investigation and close monitoring are needed on the extent of dyspnoea in different social groups, especially in the case where the pandemic is prolonged and further waves of COVID-19 appear.

## Figures and Tables

**Table 1:** Age of respondents.

Age	Number	Percentage
<26 years	50	24.5%
26–35 years	49	24.0%
36–45 years	49	24.0%
46–55 years	46	22.5%
56–65 years	7	3.4%
>65 years	3	1.5%

**Table 2:** Hospitalisation due to COVID-19.

Hospitalization Due to COVID-19	Number	Percentage
no	187	91.7%
yes	17	8.3%

**Table 3:** Severity of symptoms.

Severity of the Disease	Number	Percentage
asymptomatic	54	26.5%
oligosymptomatic	99	48.5%
fully symptomatic and severe	51	25.0%

**Table 4:** Level of dyspnoea in persons infected with the SARS-CoV-2 virus.

Shortness of Breath	$\bar{x}$	Me	*s*	Min	Max
MRC scale	0.57	0	0.94	0	4

**Table 5:** Severity of infection versus level of dyspnoea.

Severity of Dyspnoea	Severity of Infection						*p*
	Asymptomatic		Oligosymptomatic		Fully Symptomatic and Severe		
	Mean	Median	Mean	Median	Mean	Median	
MRC	0.07	0	0.45	0	1.33	1	0.0000

*p*—test probability value calculated using the Kruskal–Wallis test.

**Table 6:** Hospitalization for COVID-19 and dyspnoea.

Severity of Dyspnoea	Hospitalization Due to COVID-19				*p*
	No (*N* = 187)		Yes (*N* = 17)		
	Mean	Median	Mean	Median	
Assessment of dyspnoea	0.47	0	1.76	2	0.0000

*p*—test probability value calculated using Mann–Whitney test.

**Table 7:** Gender and level of dyspnoea.

Measure of Dyspnoea	Gender				*p*
	Female		Male		
	Average	Median	Average	Median	
Assessment of dyspnoea	0.71	0	0.37	0	0.0032

*p*—test probability value calculated by the Mann–Whitney test.

## Data Availability

The data that support the findings of this study are openly available in RepOD at https://doi.org/10.18150/2YITU6.

## References

[1] Gorbalenya A.E., Baker S.C., Baric R.S., de Groot R.J., Drosten C., Gulyaeva A.A., Haagmans B.L., Lauber C., Leontovich A.M., Neuman B.W. (2020). Severe acute respiratory syndrome-related coronavirus: The species and its viruses—A statement of the Coronavirus Study Group. Nat. Microbiol..

[2] Guan W.J., Ni Z.Y., Hu Y., Liang W.H., Ou C.Q., He J.X., Zhong N.S. (2020). Clinical characteristics of coronavirus disease 2019 in China. N. Engl. J. Med..

[3] Holshue M.L., DeBolt C., Lindquist S., Lofy K.H., Wiesman J., Bruce H., Pillai S.K. (2020). First case of 2019 novel coronavirus in the United States. N. Engl. J. Med..

[4] Dong L., Bouey J. (2020). Public mental health crisis during COVID-19 pandemic, China. Emerg. Infect. Dis..

[5] Fischer R., Bortolini T., Karl J.A., Zilberberg M., Robinson K., Rabelo A., Mattos P. (2020). Rapid review and meta-meta-analysis of self-guided interventions to address anxiety, depression, and stress during COVID-19 social distancing. Front. Psychol..

[6] Heitzman J. (2020). Impact of COVID-19 pandemic on mental health. Psychiatr. Pol..

[7] Sekowski M., Gambin M., Hansen K., Holas P., Hyniewska S., Wyszomirska J., Łojek E. (2021). Risk of developing post-traumatic stress disorder in severe COVID-19 survivors, their families and frontline healthcare workers: What should mental health specialists prepare for?. Front. Psychiatry.

[8] Yu C., Helwig E.J. (2021). Role of rehabilitation amidst the COVID-19 pandemic: A review. J. Transl. Med..

[9] Rutkowski S. (2021). Management challenges in chronic obstructive pulmonary disease in the COVID-19 pandemic: Telehealth and virtual reality. J. Clin. Med..

[10] Berliner D., Schneider N., Welte T., Bauersachs J. (2016). The differential diagnosis of dyspnoea. Deutsches Ärzteblatt Int..

[11] Wysham N.G., Miriovsky B.J., Currow D.C., Herndon J.E., Samsa G.P., Wilcock A., Abernethy A.P. (2015). Practical dyspnoea assessment: Relationship between the 0–10 numerical rating scale and the four-level categorical verbal descriptor scale of dyspnoea intensity. J. Pain Symptom Manag..

[12] Parshall M.B., Schwartzstein R.M., Adams L., Banzett R.B., Manning H.L., Bourbeau J., Calverley P.M., Gift A.G., Harver A., Lareau S.C. (2012). An official American Thoracic Society statement: Update on the mechanisms, assessment, and management of dyspnoea. Am. J. Respir. Crit. Care Med..

[13] Zhou F., Yu T., Du R., Fan G., Liu Y., Liu Z., Xiang J., Wang Y., Song B., Gu X. (2020). Clinical course and risk factors for mortality of adult inpatients with COVID-19 in Wuhan, China: A retrospective cohort study. Lancet.

[14] Chen X., Jiang Q., Ma Z., Ling J., Hu W., Cao Q., Zhang Y. (2020). Clinical characteristics of hospitalized patients with SARS-CoV-2 and hepatitis B virus co-infection. Virol. Sin..

[15] Wang D., Yin Y., Hu C., Liu X., Zhang X., Zhou S., Jian M., Xu H., Prowle J., Hu B. (2020). Clinical course and outcome of 107 patients infected with the novel coronavirus, SARS-CoV-2, discharged from two hospitals in Wuhan, China. Crit. Care.

[16] Wirth K.J., Scheibenbogen C. (2022). Dyspnoea in post-COVID syndrome following mild acute COVID-19 infections: Potential causes and consequences for a therapeutic approach. Medicina.

[17] Motiejunaite J., Balagny P., Arnoult F., Mangin L., Bancal C., d’Ortho M.-P., Frija-Masson J. (2021). Hyperventilation: A possible explanation for long-lasting exercise intolerance in mild COVID-19 survivors?. Front. Physiol..

[18] Aparisi Á., Ybarra-Falcón C., García-Gómez M., Tobar J., Iglesias-Echeverría C., Jaurrieta-Largo S., Ladrón R., Uribarri A., Catalá P., Hinojosa W. (2021). Exercise ventilatory inefficiency in post-COVID-19 syndrome: Insights from a prospective evaluation. J. Clin. Med..

[19] Mancini D.M., Brunjes D.L., Lala A., Trivieri M.G., Contreras J.P., Natelson B.H. (2021). Use of cardiopulmonary stress testing for patients with unexplained dyspnoea post-coronavirus disease. Heart Fail..

[20] Taverne J., Salvator H., Leboulch C., Barizien N., Ballester M., Imhaus E., Chabi-Charvillat M.-L., Boulin A., Goyard C., Chabrol A. (2021). High incidence of hyperventilation syndrome after COVID-19. J. Thorac. Dis..

[21] Symptoms of COVID-19. https://pacjent.gov.pl/koronawirus/sprawdz-objawy.

[22] Medical Research Council 1952 MRC Breathlessness Scale. https://mrc.ukri.org/research/facilities-and-resources-for-researchers/mrc-scales/mrc-dyspnoea-scale-mrc-breathlessness-scale/.

[23] Polański J., Chudiak A.K., Rosińczuk J. (2016). Kwestionariusze stosowane w ocenie wybranych objawów raka płuca. Medycyna Paliatywna w Praktyce.

[24] Chen T., Wu D., Chen H., Yan W., Yang D., Chen G., Ma K., Xu D., Yu H., Wang H. (2020). Clinical characteristics of 113 deceased patients with coronavirus disease 2019: Retrospective study. BMJ.

[25] Post COVID-19 Physiotherapy Programme. https://kif.info.pl/file/2021/04/hn194g862x5kq7658x6f.pdf.

[26] Ora J., Liguori C., Puxeddu E., Coppola A., Matino M., Pierantozzi M., Mercuri N.B., Rogliani P. (2020). Dyspnoea perception and neurological symptoms in non-severe COVID-19 patients. Neurol. Sci..

[27] Li X., Tian J., Xu Q. (2021). The associated factors of anxiety and depressive symptoms in COVID-19 patients hospitalized in Wuhan, China. Psychiatr. Q..

[28] Curci C., Pisano F., Bonacci E., Camozzi D.M., Ceravolo C., Bergonzi R., De Franceschi S., Moro P., Guarnieri R., Ferrillo M. (2020). Early rehabilitation in post-acute COVID-19 patients: Data from an Italian COVID-19 rehabilitation unit and proposal of a treatment protocol. Eur. J. Phys. Rehabil. Med..

[29] Tleyjeh I.M., Saddik B., Ramakrishnan R.K., AlSwaidan N., AlAnazi A., Alhazmi D., Aloufi A., AlSumait F., Berbari E.F., Halwani R. (2022). Long term predictors of breathlessness, exercise intolerance, chronic fatigue and well-being in hospitalized patients with COVID-19: A cohort study with 4 months median follow-up. J. Infect. Public Health.

[30] Sigfrid L., Drake T.M., Pauley E., Jesudason E.C., Olliaro P., Lim W.S., Gillesen A., Berry C., Lowe D.J., McPeake J. (2021). ISARIC4C investigators. Long COVID in adults discharged from UK hospitals after COVID-19: A prospective, multicentre cohort study using the ISARIC WHO Clinical Characterisation Protocol. Lancet Reg. Health.

